# Pediatricians, Well-Baby Visits, and Video Intervention Therapy: Feasibility of a Video-Feedback Infant Mental Health Support Intervention in a Pediatric Primary Health Care Setting

**DOI:** 10.3389/fpsyg.2016.00179

**Published:** 2016-02-16

**Authors:** Sergio Facchini, Valentina Martin, George Downing

**Affiliations:** ^1^Pediatric Primary Care Unit, Azienda per l’Assistenza Sanitaria n. 5 “Friuli Occidentale”Pordenone, Italy; ^2^Department of Developmental Psychology and Socialisation, University of PadovaPadova, Italy; ^3^Clinical Faculty, Pitié-Salpêtrière Hospital and Université Paris 8Paris, France

**Keywords:** infant mental health, primary care, pediatrician, prevention, video-feedback, parenting, attachment

## Abstract

This case series study evaluated the feasibility and acceptability of a behavioral/cognitive psychological intervention in a pediatric primary health care setting during standard well-baby visits. The aim of the intervention was to support caregivers’ sensitivity and mentalization in order to promote infant mental health (IMH). Four neonates from birth to 8 months were consecutively enrolled to test a short video-feedback intervention (Primary Care – Video Intervention Therapy, an adaptation of George Downing’s Video Intervention Therapy to primary care) conducted by a pediatrician. The 5 min interaction recording and the video-feedback session were performed during the same well-baby visit and in the same pediatrician’s office where the physical examination was conducted. During the study period, six video-feedback sessions were performed for each baby at different ages (1, 2, 3, 4, 6, 8 months). A series of different interactional situations were filmed and discussed: touch, cry, affective matching, descriptive language, feeding, separation and autonomy. The intervention was easily accepted and much appreciated by all four families enrolled. This study aimed to answer a dilemma which pediatric providers generally face: if the provider wishes to respond not only to physical but also IMH issues, how on a practical level can this be done? This case series study indicates that Primary Care – Video Intervention Therapy can be a promising new tool for such a purpose.

## Introduction

### Integrating Mental Health and Primary Care

The prevention of mental illness and physical disorders and the promotion of mental health and physical health are inseparable (Blount et al., 2007; Lake and Chan, 2014). Internationally there is currently widespread support to improve health care systems by effectively integrating mental health and primary care (Hunter et al., 2009; Zeneah and Gleason, 2009).

For many reasons, the integration of infant mental health (IMH) within primary health provider services seems especially strategic (Committee on Psychosocial Aspects of Child and Family Health and Task Force on Mental Health, 2009; Shonkoff et al., 2009; Kaplan-Sanoff et al., 2012; World Health Organization [WHO], 2013). First, in the developed world, virtually all young children are seen regularly from birth throughout the early years in primary health care settings. Therefore, universal approaches to screening and intervention can be applied to large populations of infants and toddlers (Shonkoff, 2003; Garner et al., 2012). Second, common problems in young children, such as sleep or feeding disturbances, aggressive behavior problems and emotional disturbances are often presented first to health care professionals. Third, most families value the relationship with their primary care providers and are comfortable in openly discussing concerns. Most families initially seek help for mental health concerns in the primary care setting. This is especially true for families from culturally, economically and ethnically diverse communities. Fourth, a number of preventive interventions have been developed using health care practitioners as providers (Lester and Sparrow, 2010; Hornstein, 2014). For all of these reasons, interest in how to apply principles of IMH to practice in primary care has grown.

### IMH and Pediatric Primary Care: Focus on Relationship

Current pediatric practice in many developed countries emphasizes the need for behavioral and developmental surveillance as part of preventive health care, which typically takes place within the context of “well-child” health visits (Weitzman and Wegner, 2015). Pediatric primary care providers (PPCPs) see young children and their families more frequently than any other health professional. Observations of parent-infant interactions during pediatric health care visits are a rich source of information regarding the relationship between the parent and infant. Such observations also provide opportunities for intervention. If the pediatrician is able give a useful form of brief, immediate help for the relationship, this could be valuable for a large number of families. Naturally in some cases, further assessment and intervention by a mental health professional would be indicated, but for these cases too, the pediatrician’s early detection of such problems would play an important role in aiding a parent to seek further help (Talmi et al., 2009).

Extensive research has demonstrated that responsive relationships with primary caregivers play a critical role in healthy social-emotional development (Tronik, 2007). The growing recognition of this significance of the parent–child relationship represents a paradigm shift for most pediatric health care professionals (Gorski, 2001).

### The Use of Video Feedback in Attachment-Based Interventions

Video-feedback is a powerful tool that is increasingly being used across a number of therapeutic modalities (Marvin et al., 2002; Dozier et al., 2006; Rusconi-Serpa et al., 2009; Bernard et al., 2012; Juffer and Steele, 2014; Steele et al., 2014). Attachment-based interventions, especially those with infants and young children, which incorporate the use of video-feedback are gaining an evidence base (Bakermans-Kranenburg et al., 2005; Fukkink, 2008). There are a number of ways in which video can be used (Guedeney and Guedeney, 2010). Usually video-feedback is part of a multimodal approach also including instruction, therapeutic counseling and other forms of parental support. Some approaches use a short series of sessions with specific themes designated for each session (Juffer et al., 2008; Cassidy et al., 2011; Powell et al., 2013). Other approaches base the choice of each session theme upon the particular case and how it is evolving (Beebe, 2003; Papousek et al., 2004; Downing, 2008; Papousek, 2011; Beebe and Steele, 2013). A meta-analysis of 81 studies, including 51 randomized controlled trials of interventions to improve maternal sensitivity, showed that even a few video-aided behavioral interventions with parents tend to be highly effective (Bakermans-Kranenburg et al., 2003).

Video-feedback can be used for simultaneous important purposes. It can greatly aid a parent to better notice and identify children’s cues. It can allow the parent to view and perhaps challenge her or his own behavior. It can help the parent better hypothesize the motivational roots of the child’s behavior, a set of abilities called mentalization (or reflective functioning). Mentalization capacity has been shown in itself to be a powerful predictor of infant–parent attachment security (Fonagy et al., 1991; Fonagy and Target, 2005; Slade, 2005a,b; Schechter et al., 2006; Salder et al., 2008; Steele and Steele, 2008; Berthelot et al., 2015).

### Video Intervention Therapy

Video Intervention Therapy (VIT) is a mentalization-based, cognitive-behavioral methodology (Downing, 2008). In addition to classical cognitive behavioral techniques it draws on mentalization and other techniques developed within VIT itself (Reck et al., 2004; Downing et al., 2008; Downing, 2015; Riva Crugnola et al., 2015).

A video of a parent–child interaction is filmed which is relevant to the problems the parent is having and the age of the child. The video can record the parent and child involved in playing, nursing or feeding, a bath, a diaper change, mealtime, a conflict or boundary-setting situation, and so on. The video is usually 5–10 min in length, and can be filmed in the practice setting or in the family home.

In the VIT session, one or both parents look at and reflect upon the video with the therapist. A six-step protocol is followed. As a session unfolds, two general types of therapeutic focus become possible (Downing, 2005; Downing et al., 2014). One is focusing on what in VIT is called the “outer movie”—that is, the visible behavior of both parent and child. The other is on the “inner movie” —that is, what the parent subjectively experienced during the interaction, and/or what the child perhaps subjectively experienced (the latter is reflected upon using mentalization techniques). Some attention is always given to the outer movie. Depending on circumstances (e.g., the goals of the session, the availability of the parent) the work with the inner movie may be woven in as well. Therapists also learn a specific set of categories, a “scanning map,” to look at a video in preparation for a session.

Typically in the session, in Step 1, the therapist shows a selected part of the video, and the patient is asked to comment. The patient or patients (e.g., a parental couple) are encouraged to share what they have found of interest in the video. Discussion based on these remarks may follow. In Step 2, the therapist points out a series of visible positive moments in the interaction seen in the video, and shares his or her reasons for regarding them as positive. Some additional discussion may take place. In Step 3, the therapist turns to and offers diplomatic, non-confrontative language for a negative pattern in the interaction. Most often only one pattern is selected. In Step 4, the negative pattern just highlighted is explored. When VIT is being done in a psychotherapy mode, this exploration is carried out in some depth. When it is being done in a coaching mode, the exploration is briefer. In Step 5, the therapist and the patient reflect together on one or more new actions that the patient can make at home. In other words, the focus moves now to the when, where, and how of behavioral change. In Step 6, the therapist summarizes the main points elaborated in the session.

Not every session proceeds in exactly this way. VIT is meant to be genuinely collaborative, with the patient’s input and thinking central to the process. As a result, a patient’s ideas now and then take a creative turn, and the therapeutic exchange veers away from its planned direction.

## Background

### Approach to the Case Series

Our case series study aimed to evaluate a community-based IMH preventive intervention named Primary Care – Video Intervention Therapy (PC-VIT). The key to the intervention was the use of a protocol from VIT (Downing, 2005). VIT can be employed either as psychotherapy, or, in a more limited manner, as mental health coaching (Tiffany, 1982; Jordan and Livingstone, 2013; Barnett et al., 2014; Whittaker et al., 2014). In this study a simplified, manualized coaching form was used. The preferential work with positive interactions during VIT session was chosen in order to facilitate its use by diverse health care professionals. The use of steps 1, 2, 5, and 6 of the VIT protocol are easier and are normally the first recommended way to start training in the VIT procedure. Moreover many other video-feedback interventions work only on positive patterns. The focus on negative patterns is not avoided in PC-VIT but provided only when parents see them and are willing to discuss such patterns. The more expert the professional is in VIT the more he or she can work on negative interactional patterns seen in the video recordings. To our knowledge this is the first study assessing a video-feedback intervention to support IMH in a primary care setting conducted by a pediatrician (the first author). We also did not find any other description in the literature of a video-feedback intervention to promote IMH core topics in a community pediatric primary care service.

Such an approach has the great advantage of allowing universal access to a preventive IMH service to all newborns attending the pediatric primary care office. Often parents of a newborn or infant, even if in great distress, do not recognize their needy condition and do not actively ask for help (Felitti et al., 1998; Lyons-Ruth et al., 2005; Shonkoff and Garner, 2012; Murphy et al., 2014). The active offer of a support intervention by a physician in a trustful setting could bypass such an obstacle. In addition, because PC-VIT is integrated into pediatric primary care and does not require home visits, it is economically quite inexpensive (Knapp et al., 2005).

In addition to the video-feedback technique, this approach was unusual in being done by a pediatrician trained in counseling techniques, psychotherapy, and VIT. In principle, however, the integration could just as well be accomplished, in the same setting, with two different health care providers. For example, a pediatric nurse practitioner (Freed et al., 2010), a developmental specialist (Mendelsohn et al., 2007), or IMH professional (Briggs et al., 2007), could carry out the video-feedback component during the same visit.

Feasibility and acceptability were the main endpoints (primary outcome) of this study: evaluating how this preventive IMH intervention could be embedded in a busy pediatric primary care service, how parents would accept and react to it, whether and how much parents would appreciate it, and what kind of drop-out rate might occur (i.e., if any family would refuse to continue with the video-feedback sessions while still continuing with the WBVs).

The intervention was offered to both primary caregivers (mother and father) with the intent to keep fathers engaged in the whole process (Scourfield et al., 2014), as usually fathers do not attend WBVs (Garfield and Isacco, 2006). Hence another endpoint was to measure how many times fathers attended the WBV sessions.

No formal assessment of therapeutic efficacy was done in this case series study. We collected only personal feedback from parents enrolled in the study. The evaluation of the effects of this intervention on the infant–caregiver relationship, on the parental mental states related to parenting, or on the infant attachment style was not an assessment goal.

Many models for using pediatric primary health care to promote child development and literacy have proven to be efficacious despite their low intensity and cost (Zuckerman, 2009). Furthermore many intervention protocols on child–caregiver relationship with video-feedback in normal and high risk populations have supported the efficacy of such approaches (Green et al., 2015; Hoivik et al., 2015). We therefore based this intervention on quite promising premises. However, a formal evaluation of the efficacy of the approach is the goal of another, separate study: one involving an appropriate number of infants with appropriate assessment scales and measures, currently underway with the collaboration of the University of Padova (Italy).

### Participants

The intervention was implemented with normal population families attending a pediatric primary care community office that was part of a larger primary care pediatric center serving more than 3000 children and their families. This pediatric group practice is part of the Italian National Health Service and provides free of charge care to children from birth up to 14 years of age.

Three pediatricians, two secretaries and two pediatric nurses work in the center. Each pediatrician serves a child population ranging from 900 to 1200 children, and each child is assigned to a specific pediatrician. Each pediatrician sees a mean of 250 sick children per month. The group practice receives 1200 phone calls each month.

Participants were enrolled during the first visit with the pediatrician and an infant ranging in age from 15 to 30 days. The first four babies registered with the pediatric service, after the beginning date of the study, were successfully enrolled in the study. All four cases were enrolled within a 30-day period between June and July 2014.

The intervention was offered to whichever family members initially came to the pediatrician’s office in order to register their newborn. Both parents (or primary caregivers) were then offered the 18-month long intervention. It was explained that the intervention would be intended to support the infant’s physical as well as psychological and relational development with video-feedback as a specific tool.

No family refused the intervention. Informed consent was obtained for filming infant–caregiver interaction and video-feedback sessions. Baseline data and medical histories of both parents and infant were obtained during the first visit. As the aim of the study was to assess feasibility and acceptability but not efficacy, no psychometric measures where obtained at the beginning or during the course of the intervention. Since high quality film recording was done at every WBV, in principle a further evaluation of the infant–caregiver relationship quality pre- and post-intervention could be done at some future moment.

One of the principal aims of the study was also to assess the practical issues involved in embedding a video-feedback intervention in a busy pediatric primary care office: how to record infant–caregiver interactions, for how long, when and how to show the film clip recorded for the video-feedback session, who to keep in the office during the session (i.e., parent with or without the infant). The following were also evaluated: the parents’ acceptance of the intervention; their reaction to the double professional role of the pediatrician (physician plus mental health practitioner); the number of intervention dropouts; and the number of times the couple attended the WBV together. Finally, we evaluated if this type of enhanced WBV reduced the total number of doctor’s or nurse’s primary care center access (i.e., reduced the quantity of visits for any reason: medical problems, queries about infant development, maternal anxiety, etc.).

### Intervention

The PC-VIT manualized protocol (unpublished document) was derived from the VIT standard procedure. The routinely scheduled WBVs in Italy occur at 1, 3, 6, 8, 12, 18, and 24 months. In support of an early relationship, for the purpose of PC-VIT intervention, two more WBVs were added at 2 and 4 months.

During every WBV there was a 15–20 min medical visit (for the physical examination, evaluation and discussion of medical problems) and a 40–45 min video-feedback intervention. In the 15–20 min medical visit a video clip (5 min) appropriate to the WBV (the setting and theme of the video recording changed with the age of the baby as explained later) was recorded (**Table 1**). The 5 min recording of interaction and the video-feedback session were performed during the same WBV and in the same pediatrician’s office where the medical examination was conducted.

**Table 1:** Brief scheme of single WBV plus PC-VIT session.

Minutes	Medical examination	Video recording	PC-VIT
0–20	x	x (5 min)	
20–40			x
40–60	x		x

*Medical examination, includes physical examination in the first part and recommendations plus indications about psychomotor development and physical health promotion advice. WBV, well-baby visit. PC-VIT, Primary Care – Video Intervention Therapy.*

Every video-feedback session was performed for each baby at different ages (1, 2, 3, 4, 6, 8, 12, 18 months). A series of different interactional situations (touch, cry, and needy behaviors, affective matching, descriptive language, feeding, separation and autonomy, book reading, limit setting) were filmed and discussed. The whole process was recorded as well: both the medical visit and the VIT procedure (for the pediatrician self-evaluation see Fukkink et al., 2011 and other assessment/analysis purposes see Meade et al., 2014). Each session had a fixed set of questions that were asked. However, the sequencing of these questions was left open, so that they could be adapted to the natural flow of the intervention.

The categories of the fixed set of questions were: (1) attachment based interaction observations and relevant probe questions, and (2) theme-specific probe questions designed to promote reflective functioning. Regardless of what was seen in the video clip these points had to be addressed. No written learning materials or pamphlets were given to families at visits to take home. Input given to the parents was limited to the discussions in the WBV sessions themselves.

As already mentioned, a helpful aspect of this intervention was providing both medical and mental health advice and support by the same practitioner, in addition to the trustful atmosphere this tends to create. Often enough, during the first years of infancy, issues concerning relationship and emotional regulation can be closely intertwined with medical issues. A frequent and typical example is the 1–3 month “infant colic.” Many parents confuse frequent infant crying as a physical issue: they imagine the crying is being caused by, e.g., “tummy ache” or “food intolerance.” And at the same time they may consider the idea of soothing the baby, directly in the caregiver’s arms, as a way of spoiling the newborn. In such instances the pediatrician has the opportunity to help them face at the same time, within the same session, both the physical issue (e.g., “infant colic”) and any related relational aspects. In a short amount of time this can provide clarification and guidance for the family. The unique conditions and emotional climate of the WBVs seem to allow parents to take such information quite rapidly, and to profit from it on a practical level, making shifts in both their thinking and their interactional behavior.

#### Case 2 Vignette: WBV 2 Months; PC-VIT Step 2

This was the first video-feedback session with this mother. She had attended a previous WBV together with her husband. This time she was accompanied by her own mother. This mother (of the infant) felt very uneasy with her infant’s crying. She reported being unable to calm her baby down and was beginning to think her baby had a physical problem which was causing such inconsolable crying. She had many doubts as well about being a good mother, and often felt anxious and incompetent.

We report here verbatim the dialog between the pediatrician (P) and the mother (M). The video clip was registered after the pediatrician had induced the baby to cry (theme of the session) using the Ortolani maneuver: a standard procedure used to check congenital hip dislocation in newborns which is a bit distressing for infants and which always makes them cry.

After the infant started crying the pediatrician left the office, leaving the mother alone. After 5 min the pediatrician returned and, after a short interval, started the PC-VIT session. Together with the mother the pediatrician looked at the just-registered recording. He paused it after 90 s, and asked the mother what she found interesting in what they had seen (procedure step 1). After this brief discussion the pediatrician showed the mother an interaction of his choosing, as reported verbatim here (step 2).

P: Let’s see another piece of the video ….

A brief (20 s) video-clip was shown where the mother picks up her crying infant, places her against her chest, and starts to swing softly while singing with a sweet tone of voice. After a few seconds the infant ceases to cry, opens her eyes and appears relaxed.

P: So this is very good. She is in distress and you are calm.
M: Mm, mm *(nodding her head, but still looking puzzled).*
P: Your tone of voice is calm, sweet. That is very good! This helps the baby to calm down.
M: Mm, mm *(nodding her head).*

The same sequence was shown again to the mother.

P: She is in distress, because something inside her is distressing her. But you have a calm tone of voice. This physical contact is also important *(the pediatrician shows this in the video to the mother)*, and the movement as well. These are two very good things you are doing.
M: Mm, mm and she likes (listening to my) singing.

The mother here became more relaxed and smiling while looking at the video clip.

P: Yes! And so little by little she is calming down. The distress could last a bit, but you are calm with her, and that is the main thing.

The same sequence, and a bit more, was shown again to the mother. The recording was stopped leaving on the screen an image of the mother singing with the infant clinging to her and looking at her with open eyes.

P: So, this experience of being distressed, and then being calmed down by you, her feeling this closeness with you, this must bring a sensation of wellness, of security inside her … and this is what will help her gradually learn to calm down by herself. Because she has had this good experience.
M: Yes, great. This is what I want to give her.

The mother was smiling and relaxed.

P: You are doing it very well. Yes.
M: I want to do the best for her.
P: You are doing very well and the crying, and the response to crying …. crying is like saying “help me, I don’t know what to do, something is happening inside me and I don’t know what … Help me to calm down.” And you are doing it with her! So she has this good experience.
M: What I really appreciate is that you give me much security. This helps me a lot.

#### Case 1 Vignette: WBV 1 Month; PC-VIT Steps 1 and 2

Before this visit there had already been a 15 days extra-WBV and another office visit with both parents. This mother was quite anxious about the needy behaviors of her infant, thinking that if she gave her baby all she was asking for she would spoil her. For the mother it seemed also emotionally difficult to tolerate both the infant’s cry and her need for body contact.

This was the first video-feedback session at the 1 month WBV. Only the mother (M) was present. The theme of the session was touch and body-to-body contact between caregiver and infant (X). For the recording, the pediatrician (P), before leaving the office, told the mother to hold her baby and to behave as she usually did at home. In the 5-min recorded film the infant first cried for 90 s on the examination table (the mother was seated beside it and the infant was lying on the table), then the mother took the infant on her lap and tried to distract her with some objects. Eventually she moved the infant to her chest, talking to her. Little by little the infant relaxed, even if not completely, with a few whimpers and body adjusting continuing. In the verbatim transcription below the pediatrician showed a 10 s interaction where the infant was beginning to relax.

P: Let’s look again at this video clip …. she was just crying, and now she’s got a few hiccups, but she is reducing her movements. She is not completely calm yet but she is relaxing little by little.
M: She’s quieter.
P: She has calmed down.
M: Yes.
P: This contact, to stay there with her mum … she likes it.
M: Yes.
P: What do you think?
M: Yes, I agree with that.
P: Ok, it appears to me this …. here look …. she gets agitated and then she calms down, but here, to hold her in your arms in this way …. Eh that movement (*one visible in the video clip)*, ok, it appears that that contact helps her to calm down, do you agree?
M: Yes *(but hesitating, and looking not really convinced).*
P: I would like to ask you now … how does it feel to hold her in your arms? This body contact... how is it for you?
M: I like it very much, but after a while I get tired, I get tired...
P: Ok, let’s talk more about the experience you have.
M: Ah, I would hold her all day long if only I could...
P: Yes?
M: Yes, yes I... when I have her against me it’s wonderful.

As this was not evident by her manner of saying it, the mother didn’t appear credible. Therefore instead of confirmation, some reflective questions followed.

P: Ok and … how do you know it? How do you understand it?
M: Eh, because I feel emotional, I cry, I look at her and I cry because my feelings …. that is, sometimes I look at her and say “have I made this baby?” and this really moves me to cry.
P: So what do these tears mean?
M: Eh, positive emotions … I don’t know, a bit of distress from my side, being tired, but after I say “poor little creature, what does she have to do with this!” I don’t know….
P: Mm mm.
M: Sometimes I get angry, I rebuke her a bit. Sometimes she gets quiet and doesn’t whine anymore, and some other times she starts...
P: Mm mm.
M: Sometimes the mother *(talking about herself)* says “I do not have to shout …. keep quiet, sit down there … because, in the end, it is useless ….”.

For the pediatrician, he felt quite positive that the mother evidently felt trustful enough to move to talking about this negative side. These were obviously topics worth further investigation and reflection.

P: So we notice here that, when X *(infant’s name)* is a bit like now *(in the video clip)* not calming down quickly, inside of you two different things happen: one is that you are happy to hold her, to feel this sensation that she is yours, that she is born, and is here.
M: Yes, yes.
P: The other is that sometimes partly you do not like it *(to hold her)*, that this does not work well for you.
M: And it is for this reason that I did not want to use the swaddling clothes … because I feel the need to separate from her.
P: Yes, mm.
M: Yes, the need that she stays for half an hour...
P: This is important for you, to feel this and being able to recognize your own needs, it’s important.
M: I say to myself “take her with you now, I’m not able...I need to calm down a bit....” But soon after that I’m missing her again.
P: Mm mm, yes.
M: But often physically I just can’t manage it.

The pediatrician further helped the mother to reflect on what was happening inside her. The still frame of the video clip was visible on the screen and both referred to it.

P: So here there are several things going on inside you.
M: Yes, I try not to fidget because I know it is bad for her, maybe she could feel it. I try but I do not always succeed.
P: I would like to show you again *(showing the video clip again)* here how this, this body-to-body contact that is happening here, this contact, and movement, are calming her.
M: She likes it very much. She likes it too when I massage her feet and tummy.

The pediatrician repeatedly emphasized the positive actions the mother made to calm her infant. To show resources and capabilities which are already present is fundamental with such fragile parents.

P: These movements we see here …. also the caressing you were doing before... we can see they calm her down, let’s see it again...
M: Yes, yes.
P: Here you see, your sweet tone of voice, she likes this calm tone of voice very much. And this soft movement in your arms, she likes it. Receiving these things, for X, is very good, it creates inside her a sensation of being calm …. All this will eventually help her learn to calm herself, at least some of the time, when she needs it. Does this make sense to you?
M: Yes, yes
P: This experience produces in her the sensation: “ok, here with mum I can experience this nice sensation of being calm and secure.”

These two case vignettes give an idea of how powerful the use of PC-VIT can be. Parents see themselves in the video clip, and they are immediately engaged. They are right away usually motivated to discuss what happens between them and their infant. Thanks to this, and together with the trusting relationship with the pediatrician, they are ready to reflect. They leave the session with clear images of their capabilities, as well as with concrete ideas about new steps they can take in their interaction with their infants.

In these two vignettes the focus was mainly on positive reinforcement and simple mentalization techniques. This is not the full picture, as other techniques, not described here, are used as well; but it should give a sense of the PC-VIT functions in a pediatrician’s office.

## Discussion

### Participants Characteristics

Four newborn babies and their families were enrolled within a 30 days period in June–July 2014 (**Table 2**). In all four cases the newborn was the first child. Only one mother had had a spontaneous abortion in the past. One baby was born by vaginal delivery and three by caesarian section. All four babies were born at term, with an APGAR score between 9 and 10 at the first minute, the mean birth weight was 3.2 kg.

**Table 2:** Infant’s characteristics.

	Gender	Previous pregnancy	Type of delivery	Gestational age	APGAR	Birth weight
Case 1	F	0	Caesarian section	38/52	10,10,10	3.05 kg
Case 2	F	0	Caesarian section	41/52	/	/
Case 3	M	0	Caesarian section	39/52	9,10,10	2.85 kg
Case 4	M	1 (abortion)	Normal vaginal	41/52	9,10,10	3.84 kg

All four couples were married. The mean age of the mother and the father was 36 years (**Table 3**). Considering the mean age of Italian parents at birth of the first child, 36 years is quite usual. In one couple, both partners were Italian, in the other three, one partner was Italian and the other from elsewhere in the European Union. Two parents had a university degree, four had a high school degree and one a middle school degree. All parents were employed in medium to high level jobs.

**Table 3:** Parent’s characteristics.

	Age		Origin		Study title	
	M	F	M	F	M	F
Case 1	33	35	EU	IT	High school	High school
Case 2	/	36	EU	IT	University	University
Case 3	36	32	IT	EU	University	High school
Case 4	40	41	IT	EU	Middle school	High school
Mean	36	36	/	/	/	/

*IT, Italy. EU, European Union.*

### Intervention Feasibility

Technological requirements to implement PC-VIT intervention are easily available to primary care professionals. All that is needed is a personal computer, a screen and a web-cam. Every professional normally uses them already or can purchase the equipment with little expense. No specific expertise is required to use such machinery.

The total number of WBVs after enrollment, irrespective of case number and infant age, was 20. In all but two (18/20 = 90%) a VIT session was carried out (**Table 4**). On one occasion the recording was not made because of an equipment problem, and on the other occasion parents were so worried about a medical problem (infant’s urinary tract infection) that the whole visit was dedicated to this issue. Video recordings were easily realized during WBVs in the pediatric office context.

**Table 4:** Pediatrician’s office access, WBV and PC-VIT sessions.

	Case 1	Case 2	Case 3	Case 4	Total
Total n° WBV	6	5	6	6	22
N° WBV after enrollment	5	4	6	5	20
N° PC-VIT session	5	3	5	5	18
Mother/Father	1	3	1	3	8
Mother/Grandparent	0	1	2	1	4
Mother	4	0	3	2	9
Doctor’s office visits	2	2	3	2	9
Nurse’s visits	3	0	1	0	4
Phone calls	3	2	5	1	11

*WBV, well-baby visit. PC-VIT, Primary Care – Video Intervention Therapy.*

The 5 min recording always provided sufficient interaction examples for a productive exploration during the VIT session, both positive and negative elements could easily be seen. For example, most of the time in the first 60 s of the recording there was a good example of a positive interaction which could be reflected on. It was rarely necessary to continue watching the recording for more than 2 min in order to find brief moments of interest.

It is important here to bear in mind also that the pediatrician himself, when working with the recording, was seeing it for the first time. This is an unusual prerequisite of this intervention, essential for the WBV setting. Normally in other forms of video-feedback intervention the video recording is made on one occasion, either by the practitioner or the family themselves, and then the intervention takes place on a second occasion. This allows the practitioner to have at some point a moment alone with it, for preparation. However, in this case series the need for the pediatrician to comment at once on the video, immediately after having seen it, was not a problem. Likely this, in part, reflects the reality that the types of interaction filmed are ones with which any pediatrician who does WBVs is highly familiar.

For all these reasons PC-VIT appears to be a feasible intervention in the primary care pediatrician’s office. No major technological, time-consuming procedures or other obstacles seemed to jeopardize the intervention. Five minutes of video recording during WBV were well accepted by parents and provided good material for video-feedback.

### Intervention Acceptability

All families to whom the intervention was offered accepted being enrolled in the study and signed the informed consent of the intervention protocol and a specific consent form for recordings (recruitment rate 100%). All families (at least one parent each WBV) attended all sessions (attendance rate 100%) and there were no drop-outs.

In 8/20 sessions (40%) the father was present; the main reason for fathers skipping the appointment was work obligations. In Italy maternity leave starts before delivery and lasts a minimum of 5 months; the majority of maternity leaves are taken by mothers even if fathers are allowed to take them as well.

The general approach of the pediatrician in observing the infant–caregiver interaction together with parents in order to “help the baby to develop” was easily accepted by parents without their seeming to feel under investigation or being judged. On the other side, even in this small case series sample, three out of eight parents had to overcome an initial reluctance about looking at themselves in the video. In only one case (a father) was this a genuine obstacle to the VIT session. In the other two cases this sense of vulnerability was easily overcome, perhaps due to their comfortable relationship with the pediatrician.

The intervention seemed to be very well accepted by parents (high attendance rate and no drop-outs) and seemed to respond to an often unmet need of discussing with a professional about not only physical or developmental issues, but also about emotional and relational aspects of parenthood. In this regard PC-VIT appears to be an adoptable intervention by primary care services in response to this important parental need as shown during the final parental semi-structured interview about their PC-VIT experience.

### Preliminary Outcomes

Considering the activity of the pediatric office as a whole for all four cases, during the 8 months of the study, nine physician examinations for medical issues (mean of two visits per infant), four nurse’s visits, and eleven phone calls for advice from nurses were performed. This number of contact with the pediatric group practice appears to be less than average. This is the only measurable effect we can analyze based on the data available.

As already pointed out, no formal assessment of the VIT sessions’ effect on infant–caregiver interaction, nor on parental mentalization, nor infant attachment style were carried out in this case series as this was not our aim. Many different aspects of the infant–parent relationship and of infant development could in principle be examined in this 0–18 month intervention design. We consider this a next step to be undertaken in a new project.

### Final Parent’s PC-VIT Evaluation

We here transcribe verbatim two short comments from mothers and fathers during the 8 months WBV after at least three PC-VIT sessions. Parents were asked to comment freely on what seemed different about using film recordings of themselves interacting with their infants.

#### Case 3 PC-VIT Protocol Evaluation: 8 Months WBV

Only the mother (M) was present. This family had had 5 PC-VIT sessions recorded, one of which the father was present, and in two of which the grandmother attended. The mother’s comments here refer to the 8 months PC-VIT session where the theme was “separation and autonomy.” In the recording that was used for video-feedback the infant (X) was seated in a high chair with mother directly facing him. The pediatrician left the office, leaving them interacting, then he knocked on the door and the mother went out of the room. The infant was thus left alone for 1 min, after which the mother reentered the room. This is our standard filmed activity for the 8 months WBV.

P: I would like to ask you something else. What we have done so far with the video … Do you see a difference between talking about X (*infant’s name)* and what happens between you two, compared to watching what happens between you and talking about it?
M: Ehh, it is completely different!
P: Ok...
M: Because to look at oneself in this way, I mean when I am with him I see only him, I don’t see anything else. Instead here *(in the video session)* I can see us together, eh, then you can pick up many different things like the complicity between our gaze, it was there, wasn’t it? Instead you can take it for granted and not pause to reflect on the moment of reunion … no? Regarding the need for closeness... and it’s nice to see that anyway you give him tranquility (comfort)... because here you see him when he’s alone and how he behaves and then, when I come back in the room, you can see how he reacts and shows a different behavior, alone I would not have noticed it... in the video his reaction is very clear.
P: And … what we do here with the video, in this way, for half an hour … When you are back home, do you take something with you? I mean these images, and our discussions … do you bring these home with you, inside yourself, and do you use them a little back at home?
M: Yes, yes. Hee … obviously they make me reflect. Because, for example, when he cries “oh I’m fed up! Here he is again!” and you are finishing your duties, and then you understand and reflect that he is crying because …. because he needs contact, he needs contact to calm down, so, instead of letting him cry for 10 min alone, you go there a moment, try to calm him down, you try to think about it, try to calm him down and then you go and finish your duties.
P: So what you are saying now is that you are starting to better understand his point of view?
M: Yes *(with the video)* you understand his point of view more.
P: Yes, his state.
M: Yes.
P: Well, eh … I think this is relevant.
M: Yes, and also the fear. Last time we reflected on fears, why babies cry, because many times it seems like a whim to the parents, because “you are changed, you are clean, you have eaten, you are in your cute room softly laid down, but what more do you want? Leave your mother in peace for 5 min!” and instead he is afraid, to be left alone and so you think about it and this gives you ….
P: the motivation to go and calm him down, is it so?
M: Yes, yes … to me this thing *(video-feedback sessions)* has been very useful.
P: Nice to hear this from you.

#### Case 4 PC-VIT Protocol Evaluation: 8 Months WBV

Only the mother (M) attended this session. This family had 5 PC-VIT sessions, in three the father was present, and in one the maternal grandmother. This couple was very attuned; unfortunately the father, sensitive and caring, was, however, not present in this session to give his feedback.

P: According to you is there any difference between doing what we do here: looking at the video clip and talking about it, compared to talking about interaction and relationship without watching the film recordings?
M: Eh, quite a lot according to me, because by watching I understand more, I am able to see little moments, and to pick up little things, if we had not watched the video I would not have been able to understand ….like I do now: the 2 s reactions...
P: Mm mm, ok.
M: … of sadness and then immediately after of joy …. I think it is very useful.
P: Ok and in what sense is it more useful according to you?
M: To help the mother to understand her behavior, if there really is a good relationship between mother and infant.
P: Mm mm, ok.
M: Things like that, I believe it is really …. Good.

## Concluding Remarks

In many countries today WBVs now make a significant contribution to early physical health and development. What our experience with PC-VIT suggests is that this same setting offers an additional valuable opportunity (Dozier and Bick, 2007; Mendelsohn et al., 2011a,b; Buchholz and Talmi, 2012; Ordway et al., 2015). Parents can be assisted, in a gentle, supportive manner, to think in new ways about the parent–infant relationship and about their infants’ inner life. Parents usually arrive at WBVs already wanting to receive information and help (Olson et al., 2004). Many of them openly show, with the pediatrician, an immediate level of trust which in other professional contexts (social work, day care, etc.) would typically take longer to emerge.

There is of course a second advantage in adding such an approach. In the event that any parent–infant dyad appears to have extreme difficulty, then this parent could be immediately encouraged to seek out more extensive therapeutic help (Weitzman and Wegner, 2015). As much developmental and clinical research has shown, the earlier such aid is provided, the better (Gilbert et al., 2009; MacMillan et al., 2009). However, finding such dyads, and motivating them to seek additional assistance, is only a secondary purpose for PC-VIT. Its primary purpose is preventive, as well as reaching a much larger number of families.

No doubt that the four families of the study strongly appreciated this intervention. There was an attendance rate of 100% and no dropouts. The attendance rate of fathers was also up to 40% of WBVs, more than the normal attendance rate of fathers at WBVs (an effect which perhaps could be strengthened further). Every parent answered a semi-structured interview evaluating their experience and perception of the intervention (Ayala and Elder, 2011). As depicted in the parents’ final comments vignettes, the overall valuation of the WBVs with PC-VIT session was notably positive. All participants valued the intervention as useful to them in enhancing the ability to understand their behaviors and their infant’s behaviors better. They reported feeling more confident as parents and more aware of their capabilities.

In regard to feasibility and adoptability of this intervention to other pediatricians, to different settings (not only pediatrician’s office) and to a larger population some considerations can be proposed bearing in mind that only a new study can answer the many questions that this case study of four families raises.

PC-VIT approach requires a basic technological equipment, easily attainable and usable in the same office where WBVs take place. All the procedures are quite inexpensive. The practicality of this protocol did not show any major drawbacks during all the 20 video-feedback sessions performed during the study. The two main difficulties to translate in practice and disseminate (adoptability) this approach would seem to be: time expenditure and primary care professional’s training.

If we consider, for example, the Italian National Health System, an average primary care pediatrician (completely funded by government) takes care of one thousand children. In such a case there will be a turnover of nearly ten children a month with ten new newborns registered. In this case series we considered all WBVs completed up to only the 8th month visit, as the study is not yet completed, but the complete intervention protocol includes WBVs up until the 18th month WBV. Providing each newborn nine PC-VIT sessions in the first 18 months could increase the pediatrician’s workload by 1-2 h per day.

A way to reduce such a workload could be by cutting the total number of PC-VIT sessions (i.e., 2 and 4 months WBV). Selecting parents and families that need PC-VIT intervention with the use of questionnaires, for example, self-administered or professionally administered, might conceivably be one way of selecting who to target. Or one might envisage contexts in which PC-VIT might be used for specific problems and developmental issues, e.g., difficulties with breastfeeding and feeding, excessive crying, or the like. One to three PC-VIT sessions might perhaps serve as a first level of assistance, with a referral for more extensive help being made only when necessary. Again, the thought here is that WBVs provide the possibility to catch such problems early, offering an opportunity to prevent development of a worse or more chronic difficulty.

An alternative could be to add a mental health professional beside the pediatrician, in the same office. Both professionals could work as a team, each one with different objectives, but working together in the same office and at the same time. There is a long standing similar project, even if with different intervention targets, with a lot of research to support the efficacy (Mendelsohn et al., 2007, 2011a,b).

However there is currently insufficient evidence to determine the optimal timing and intensity of primary care interventions to promote IMH (Guedeney et al., 2011). Our expectation is that the small amount of extra time required for a video intervention will produce measurable positive results in interaction quality, parental mentalizing, and infant attachment (Bakermans-Kranenburg et al., 2003) as already proven in other video-feedback intervention projects. Our expectation is that these results will prove to be empirically measureable. As mentioned, research on PC-VIT currently underway at the University of Padova (Italy) will hopefully shed more light on these questions.

Structured home visiting programs are of course another means of providing early preventive help (Olds et al., 2007; Juffer et al., 2008), but obviously pediatric primary care interventions can be undertaken at a much lower economic cost. In any case, data-supported cost-effectiveness analyses will be needed to better understand implications for public health policy (Robling et al., 2016). Likely such analyses will especially clarify whether pediatric interventions should be used for the population as a whole or for specific at-risk populations.

As for training of pediatricians or related professionals (e.g., pediatric nurses, IMH professionals, early childhood practitioners, health visitors, child care professionals), it is yet to be worked out how this can best be done (Korfmacher, 2014). We anticipate that such training can be relatively brief, since what must be taught is a simplified coaching variant of VIT, utilizing only several easily mastered techniques. Based on the third author’s considerable experience teaching VIT coaching in other contexts, it seems reasonable to assume that PC-VIT can be quickly learned. Primary care professionals are today aware of the striking importance (Shonkoff, 2003; Shonkoff et al., 2009; Shonkoff and Garner, 2012) of early life preventing intervention targeting the infant’s social environment and they seem interested and keen to train in this regard.

The methodology of Antoine Guedeney, creator of ADBB, the Alarm Distress Baby Scale (Guedeney and Fermanian, 2001) has inspired us. The ADBB is a screening system of observation for infant social behavior, designed to be taught to pediatricians and other PPCPs and to be utilized by them in WBVs (Puura et al., 2010; Burtchen et al., 2013). A quite limited instruction period (2 weekends) has proven sufficient for training pediatricians in the system. ADBB has now been adopted in a number of different countries. Extensive research has been carried out, showing that wherever it is taught, the number of referrals of troubled parent–infant dyads by PPCPs to mental health services noticeably increases (Dollberg et al., 2006; Guedeney et al., 2013; Matthey et al., 2013).

We find encouraging how Guedeney’s results have demonstrated the rapidity with which PPCPs can learn a new mode of evaluating infant social behavior. The difference between the ADBB mode of live observation and our mode of video observation is quite small. Some other training projects for primary health care practitioners, aiming to teach how to support parent–infant interaction, have also required only limited amounts of training (Layiou-Lignos et al., 2005). Clearly optimism seems warranted in this regard.

The applicability of PC-VIT protocol to other primary care professionals needs to be further assessed. Here only one pediatrician tested PC-VIT in his office, but as many other similar video-feedback interventions in primary care demonstrate, they can be easily implemented in many different settings by professionals with different expertise.

The first author has already trained some pediatric primary care professionals of his region in the use of PC-VIT. No data are available but unstructured interviews with them showed their strong interest in implementing this procedure. Our anecdotal observation is also that some pediatricians that started integrating PC-VIT with WBVs: reported that once they have learned and started using PC-VIT, it also aided them during times when the video was not involved. On the one hand, they noticed much more with respect to the live parent–infant interactions taking place in the office; on the other hand, they felt they had a better repertoire of responses to parental questions and concerns, for example questions about touch on issues similar to those described above. These additional side effects also seem unsurprising, even if here too it does not reflect a primary purpose. The adoptability of the PC-VIT procedure by primary care professionals therefore seems promising.

Plenty of open questions remain. Should PC-VIT ideally be done with all parents, or with selected subgroups? And if with subgroups, then which ones and determined how? Concrete data about the tradeoffs of time cost vs. developmental benefit are needed. When benefits are found, for which types of parent and/or infant do they seem most prevalent, and with what effect sizes? What might this tell us about how wide a net should be cast? A lot more information is needed, but so far the prospect of adding PC-VIT to WBVs looks promising.

## Author Contributions

SF developed the project, the adaptation of Video Intervention Therapy to the Primary Care setting and was the only professional performing the video-feedback sessions (he is a pediatrician, psychotherapist and VIT teacher and supervisor). VM was responsible for the data collection/handling, the review of the literature and the drafting of the manuscript. GD supervised the design of the study, the development of the PC-VIT manual, and revised the content critically. He also supervised some PC-VIT sessions.

## Conflict of Interest Statement

The authors declare that the research was conducted in the absence of any commercial or financial relationships that could be construed as a potential conflict of interest.

## Abbreviations

IMH: infant mental health
PC-VIT: Primary Care – Video Intervention Therapy
PPCP: pediatric primary care provider
VIT: Video Intervention Therapy
WBV: well-baby visit
